# Can Catch-Up Vaccinations Fill the Void Left by Suspension of the Governmental Recommendation of HPV Vaccine in Japan?

**DOI:** 10.3390/vaccines10091455

**Published:** 2022-09-02

**Authors:** Asami Yagi, Yutaka Ueda, Satoshi Nakagawa, Sayaka Ikeda, Mamoru Kakuda, Kosuke Hiramatsu, Ai Miyoshi, Eiji Kobayashi, Toshihiro Kimura, Taichi Mizushima, Yukio Suzuki, Masayuki Sekine, Kei Hirai, Tomio Nakayama, Etsuko Miyagi, Takayuki Enomoto, Tadashi Kimura

**Affiliations:** 1 Department of Obstetrics and Gynecology, Osaka University Graduate School of Medicine, Osaka 565-0871, Japan; 2 Division of Cancer Statistics Integration, Center for Cancer Control and Information Services, National Cancer Center, Tokyo 104-0045, Japan; 3 Department of Obstetrics and Gynecology, Yokohama City University School of Medical, Yokohama 236-0004, Japan; 4 Department of Obstetrics and Gynecology, Niigata University Graduate School of Medical and Dental Sciences, Niigata 951-8510, Japan; 5 Clinical Psychology, Graduate School of Human Sciences, Osaka University, Osaka 565-0871, Japan; 6 Division of Screening Assessment and Management, Center for Public Health Sciences, National Cancer Center, Tokyo 104-0045, Japan

**Keywords:** Japan, HPV vaccine, cervical cancer, suspension of recommendation, catch-up vaccination

## Abstract

In 2013, the Ministry of Health, Labor, and Welfare (MHLW) in Japan announced a suspension of the governmental recommendation for routine HPV vaccinations. In 2020, MHLW started individual notifications of HPV vaccine to the targeted girls. In April 2022, the governmental recommendation was restarted, and catch-up vaccinations started. We evaluated the benefits and limitations of the MHLW’s new vaccination strategies by estimating the lifetime risk for cervical cancer for each birth FY under different scenarios to suggest a measure for the vaccine suspension generation. It was revealed that catch-up immunization coverage among the unvaccinated must reach as high as 90% in FY2022, when the program begins, in order to reduce the risk of the females already over the targeted ages to the same level or lower than that of women born in FY1994-1999 who had high HPV vaccination rates. For women whose vaccination coverage waned because of their birth FYs, strong recommendations for cervical cancer screening should be implemented.

## 1. Introduction

In Japan, public subsidies started for HPV vaccinations of females 13–16 in the fiscal year (FY) 2010. In 2013, the national program was stated for the girls aged 12–16 [1]. However, media reports of ‘diverse symptoms’ occurring after vaccination began circulating. By June 2013, the Ministry of Health, Labor and Welfare (MHLW) announced a ‘temporary suspension’ of its recommendation for HPV vaccination. These circumstances led to a sharp decline in the vaccination rate, and seven years passed with the vaccination program almost at a standstill. In October 2020, MHLW issued a nationwide notice that local governments should begin sending ‘HPV vaccine information’ to individually targeted females and parents. Finally, in November 2021, MHLW announced the termination of their ‘temporary suspension’, issuing a nationwide notice to local governments to implement ‘active vaccination recommendations’, targeting girls born in or after FY2006, beginning in April 2022 [2]. Table 1 shows the events in Japan’s HPV vaccination efforts.

The age-adjusted incidence of cervical cancer among Japanese women began increasing after around 2000, with the incidence peak shifting to younger ages [3]. Therefore, great expectations were placed on the prevention of cervical cancer by the HPV vaccine. However, Japan’s introduction of the HPV vaccine has failed. The dramatic decrease in HPV vaccinations among girls born in the birth cohorts from FY2000 to FY2005 (C. Vaccine-suspension generation in Table 1) has left thousands of young women vulnerable to cervical cancer. Although the most appropriate timing of vaccination was missed, in order to ‘ensure fair vaccination opportunities’, the MHLW announced that ‘catch-up vaccinations’ would be offered for three years, from April 2022 to March 2025, to females born in FY1997 to FY2005 who became eligible for routine immunization, while the recommendation was being suspended [4]. In addition, females born from FY2006 to FY2007 would be gradually included in the catch-up vaccination program if they surpassed their age limit for routine vaccination. However, it is unknown how well these strategies will reduce the negative impact of Japan’s nine-year vaccine hiatus. Herein, we strive to predict some of the outcomes. Using updated data, we estimated the relative lifetime risk for cervical cancer incidence and death by a woman’s FY of birth. We evaluated how much their risk was likely to be reduced by a resumption of the vaccine recommendation and by implementing a catch-up vaccination program using multiple hypothetical values for vaccination coverage to understand the expectations and limitations of catch-up vaccinations. To predict the potential outcomes of various catch-up vaccination scenarios, we used values for ‘vaccination intention’ obtained from a related internet survey as ‘estimation substitutes’. The number of vaccine doses required was also estimated based on the survey’s ‘HPV vaccination intention’ responses.

## 2. Materials and Methods

### 2.1. Cumulative Initial HPV Vaccination Rate by Birth FY

HPV vaccination rate information was derived from Japan’s MHLW Welfare Science Council’s Vaccination and Vaccine Subcommittee Side-Reaction Study Group, the implementation report of the emergency promotion project of HPV vaccination from FY2010 to FY2012 and the data of the regional health promotion program report from FY2013 to FY 2019 [5]. These statistical data had a problem in the statistical methods, such as dividing the inoculation number into 2 grades, one of which is not the target age for vaccination. In our previous report, we estimated corrected vaccination rates for each birth FY after 2002 by using calculate model correcting this point [6]. The corrected cumulative initial HPV vaccination rates were as follows: 0.4% (2002), 0.2% (2003), 0.1% (2004), and 0.0% (2005). We newly estimated the following grade-specific vaccination rates for each birth FY: Birth FYs up to 2003: 6th–10th grades; Birth FY 2004: grades 6th–9th grades; Birth FY 2005: 6th-8th grades; Birth FY 2006: 6th–7th grades; and Birth FY 2007: 6th grades. The following vaccination rates for FY2020, which were not available in the national data, were calculated using data obtained from 13 municipalities in the Osaka Prefecture and population data from the National Population Census: Birth FY 2004: grades—10th grade; Birth FY 2005: 9th grade; Birth FY 2006: 8th grade; Birth FY 2007: 7th grade; and Birth FY 2008: 6th grades. When there was no resumption of governmental recommendation or catch-up vaccination, and only individual notification provided by mailing, we assumed that the vaccination rates for the same grades for those born after FY 2005 would be the same.

### 2.2. The Relative Lifetime Risk of Cervical Cancer Incidence and Death

Values of relative lifetime risk for cervical cancer incidence and death for each birth FY were calculated according to the methods used in our previous studies, assuming a risk of incidence and death of 1.00 for females born in the totally unvaccinated baseline FY of 1993 [7,8] (Supplementary Table S1). Data from the Japanese Family Planning Association was used for the sexual experience rate, as follows: age 12: 0%, age 13: 1%, age 14: 2%, age 15: 5%, age 16: 15%, age 17: 25%, age 18: 42%, age 19: 55%, age 20: 66%, age 21: 72%, age 22: 75%, and lifetime: 85% [9].

We calculated how future cervical cancer incidence and death rates might increase with decreasing vaccination rates by FY of birth, depending on vaccination rates reached after FY2022. The differences between the relative risk of incidence and death for each birth FY were calculated using an assumption of routine and catch-up vaccination rates and the risk for each birth FY—if the relative risk for females born in FY1999 continued for females born after FY2000. The differences were multiplied by the number of incidences and deaths per year. The number of lifetime incidences and deaths from cervical cancer for each birth FY was assumed to be the same for each age group per given year. The most recent data, 10,978 cases in 2018 and 2921 deaths in 2019, were used [10,11].

The alternative scenarios used are as follows: In the first, there was no resumption of governmental recommendation nor introduction of catch-up vaccinations, and only individual notification by mail was provided. In another scenario, the vaccination rates from catch-up and routine vaccinations were spread evenly from FY2022 to FY2024, and the rates for females non-vaccinated before FY2022 reach 50% and 90%, for catch-up and routine vaccinations, respectively, in FY2024. In the last scenario, the rates for catch-up and routine vaccinations spread all at once, and the rates for females non-vaccinated before FY2022 reach 50% and 90%, for routine and catch-up vaccinations, respectively, in FY2022 [7,8]. 

For currently unvaccinated females, who were born from FY1997 to FY2004, their lifetime cervical cancer incidence and death risks for each birth FY was estimated from representative ‘vaccination intention’ survey data. These women are the targets for catch-up vaccinations.

### 2.3. Estimated Number of Vaccine Doses Required, Based on Vaccination Intentions

The number of HPV vaccination doses required to provide protection (three per girl) was calculated using the specific ‘vaccination intention’ of females targeted for catch-up vaccination. We used the following estimated catch-up vaccination rates, concentrated in FY2020, for each birth FY. The percentage of respondents who would ‘definitely like to be vaccinated’ was 22.82% (1997), 13.59% (1998), 11.65% (1999), 18.93% (2000), 24.27% (2001), 19.42% (2002), 17.48% (2003), and 16.50% (2004). The percentage of respondents who would ‘more like to be vaccinated than not vaccinated’ was 20.39% (1997), 22.82% (1998), 21.84% (1999), 21.84% (2000), 25.24% (2001), 25.24% (2002), 25.73% (2003), and 25.24% (2004). It was assumed that for females born in FY2005 and thereafter, the intention to get vaccinated would be the same as for those born in FY2004. The number of required doses was calculated by multiplying the number of expected recipients by three doses. 

### 2.4. Informed Consent and Ethical Approval

Informed consent was obtained by an opt-out method. This study was approved by the ethics committee of the Osaka University Hospital. Information for opt-out was posted on the website of the local governments.

## 3. Results

### 3.1. Cumulative Initial HPV Vaccination Rate by Birth FY

**Figure 1:**
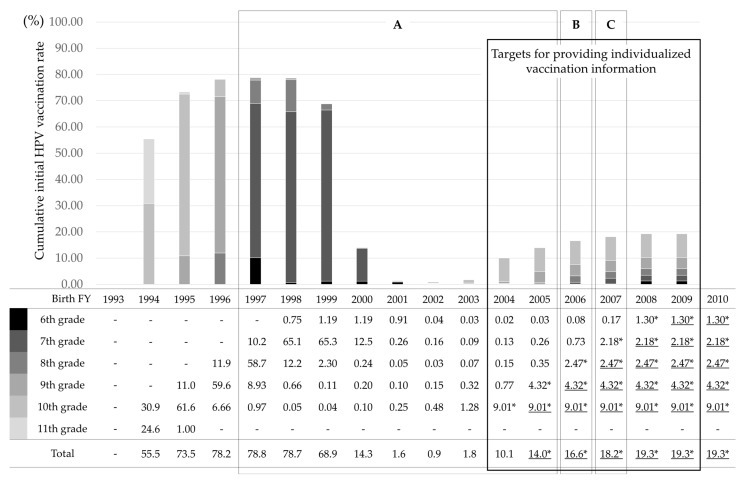
Cumulative initial HPV vaccination rate by birth year and estimated HPV vaccination rates when there was no resumption of governmental recommendation nor catch-up vaccination, and only individual notification provided by mailing. *: Calculated from a survey of municipalities in Osaka Prefecture and the national census. Underline: HPV vaccination rate of the first dose used for risk calculation when there was no resumption of governmental recommendation or catch-up vaccination, and only individual notification provided by mailing. A. Targets for catch-up vaccination targets FY2022 to 2024. B. Targets for routine vaccination in FY2022 and catch-up vaccination in FY 2023 to 2024. C. Targets for routine vaccination in FY2022 to 2023 and catch-up vaccination in FY2024.

In the present analysis, the vaccination rates by FY2019, were updated as follows: 0.9% (2002), 1.8% (2003), 1.1% (2004, total for grades 6 through 9), 0.6% (2005, total for grades 6 through 8), 0.8% (2006, total for grades 6 through 7), and 0.2% (2007, rate for grades 6) (Figure 1, Supplementary Table S2). The low HPV vaccination rate that occurred in FY2013, when MHLW’s recommendation was suspended, persisted until at least FY2019. As of FY2020, when the mailing of individual notifications was restarted, cumulative vaccination rates per birth FY were as follows: 10.1% (2004, up to 10th grade), 5.0% (2005, up to 9th grade), 3.3% (2006, up to 8th grade), 2.4% (2007, up to 7th grade), and 1.3% (2008, up to 6th grade). 

In FY2019, the vaccination rate for 10th graders born in FY2003, who turned 16-years-old while the recommendation suspension was still in effect, and individual notification had not yet started, was 1.28%. This is in contrast to FY2020, when the vaccination rate for 10th graders (born in FY 2004) rose to 9.01%, a seven-fold increase attributed to the mailing of individual notifications. 

### 3.2. Estimated HPV Vaccination Rates and Lifetime Relative Risk of Cervical Cancer without Resumption of Governmental Recommendation Nor Catch-Up Vaccination, in a Scenario Where Only Individual Notifications Were Provided and Calculated the Effects on Lifetime Relative Risk for Cervical Cancer Incidence and Death

**Figure 2:**
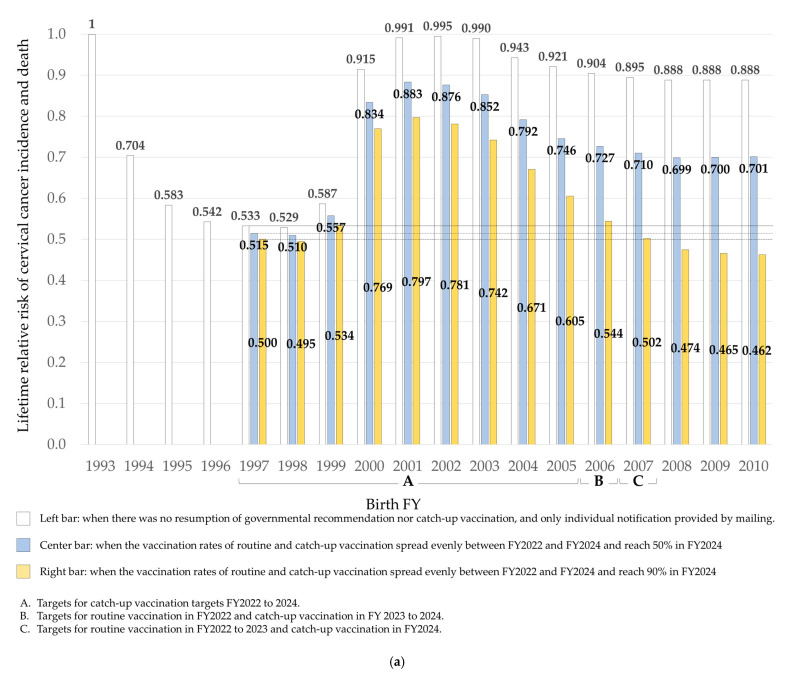

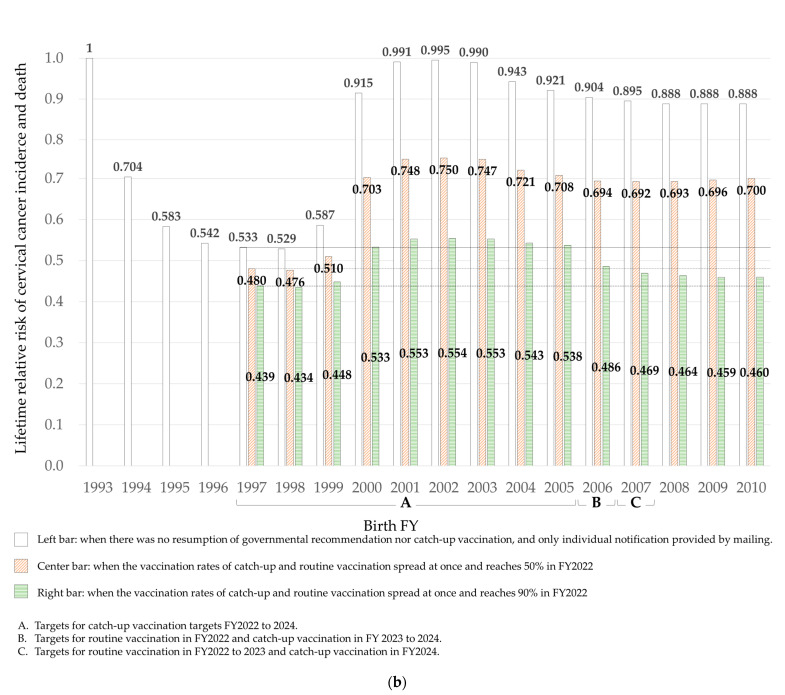

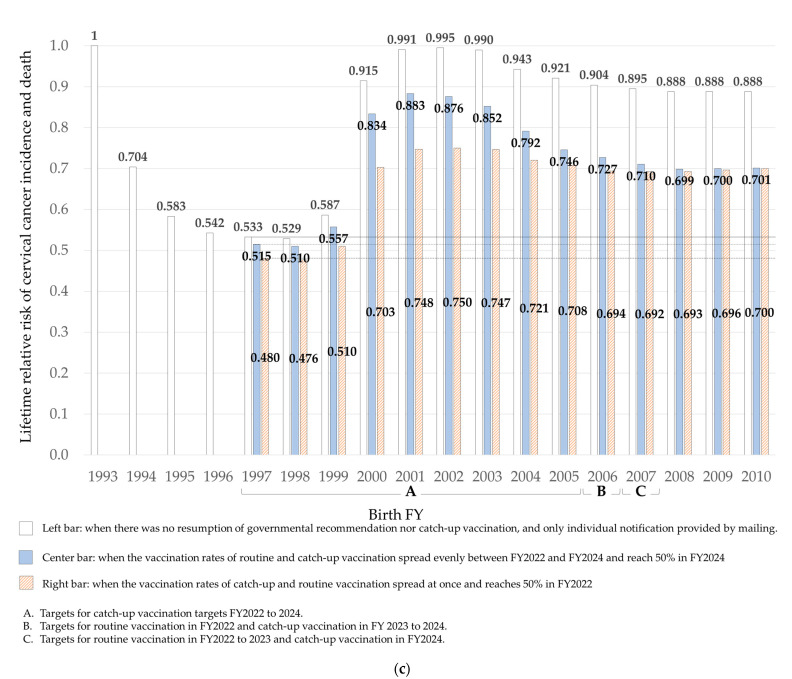
Lifetime relative risk of cervical cancer incidence and death. Vaccination status by birth FY. (**a**) Lifetime relative risk of cervial cancer incidence and death: In a scenaria where the rates of routinne and catch-up vaccinations spread evevly between FY2022 and FY2024. (**b**) Lifetime relative risk of cervial cancer incidence and death: In a scenaria where the rates of catch-up and routine vaccinations. (**c**) Comparison of lifetime relative risk of incidence and death when the vaccination rate reachees 50% in FY2022 and FY2024.

We estimated what the vaccination rates might be under a worst-case scenario where there was no resumption of governmental recommendation nor catch-up vaccination. We looked at a scenario where only individual notifications were provided. Results are shown in Figure 2.

In Japan, women born in FY1997 had the highest HPV vaccination rate ever recorded (~70%), whereas females born four years earlier, in FY1993, passed through their vaccine eligible years before the establishment of public subsidies. When the relative lifetime risks of the two groups were compared, the FY1997 women had a risk of 0.533 relative to the risk of 1.000 defined for the FY1993 women (Figure 2a).

On the other hand, the risk for females born in FY2000-2003 was 0.915 to 0.991; these women had exceeded the age for routine vaccination without ever being sent an individual notification (Figure 2a). The risks for females born from FY 2004 to FY 2010, who received individualized vaccine information, were 0.943 to 0.888 (Figure 2a).

### 3.3. Lifetime Relative Risk of Cervical Cancer Incidence and Death: In a Scenario Where the Rates of Routine and Catch-Up Vaccinations Spread Evenly between FY2022 and FY2024

In a scenario where the vaccination rate reaches 50% in FY2024, the risks for females born from FY2000 to FY2010 remain high; for FY2000 to FY2003, risks are estimated to be 0.834 to 0.852, and for females born from FY2004 to FY2010, the risks are 0.792 to 0.701 (Figure 2a). Those were higher than 0.515, the risk for the highly vaccinated FY1997 women.

In a scenario where the vaccination rate would reach a lofty 90% in FY2024, the risks are 0.502 to 0.462 for those born in FY2007 or later, which is lower than for girls of the ‘vaccinated generation’ of FY1994-1999 (Figure 2a). In both scenarios, the risks were only slightly reduced for women born from FY1997 to FY1999 who were eligible for catch-up vaccination (Figure 2a).

### 3.4. Lifetime Relative Risk of Cervical Cancer Incidence and Death: In a Scenario Where the Rates of Catch-Up and Routine Vaccinations Spread All at Once

If vaccination rates reach 50% this year (FY2022), the risks for females born in FY2000-2003 would be 0.703 to 0.747, and from FY2004 to FY2010, 0.721 to 0.700 (Figure 2b). In other words, risks could be reduced to levels similar to that of females born in FY1994, when public subsidies were started. If the vaccination rate reaches 90% in FY2022, risks will be even lower than for females born in FY1994 to FY1999, the ‘vaccination generation’ (Figure 2b). In both scenarios, the risks were reduced to some degree for the currently unvaccinated females in the FY1997-1999 group eligible for catch-up vaccinations (Figure 2b).

### 3.5. Comparison of Lifetime Relative Risk of Incidence and Death When the Vaccination Rate Reaches 50% in FY2022 or FY2024

The cervical cancer risk for females unvaccinated before FY2022, who achieve a surge vaccination rate of 50% in FY2022, would be lower for all birth FY than if the immunization rate is spread evenly across three years, only reaching 50% by FY2024 (Figure 2c). The reduction in risk for females born in the ‘vaccine-suspension generation of ’FY2000-2005 was particularly large (Figure 2c).

### 3.6. Estimated Future Cervical Cancer Incidence and Death Depending on HPV Vaccination Rates Reached in and after FY2022 under Different Scenarios

We estimate that the future incidence and deaths from cervical cancer would have been 46,719 and 12,431, respectively, for females born from FY1997 to FY2010, if HPV vaccine information is provided by individual mailings and catch-up vaccinations had not both been started (Table 2). If catch-up vaccinations were spread evenly through the next three years, to FY2024, and vaccination rates reached 50%, the incidence and death among females born in FY1997 to FY1999 would be reduced. If immunization uptake occurs all at once in FY2022 and reaches 50%, the incidence and deaths among females born in FY1997 to FY1999, and in FY2000 to FY2010, would be further reduced. However, for females born from FY2000 to FY2005, the estimated rates for incidence and death did not significantly improve—even if their vaccination rate were to reach 90% in FY2024.

### 3.7. Lifetime Relative Risk of Cervical Cancer Incidence and Death: Risks Estimated by Substituting the ‘Vaccination Intention’ Obtained from the Internet Survey as the ‘Future Vaccination Rate’

**Figure 3:**
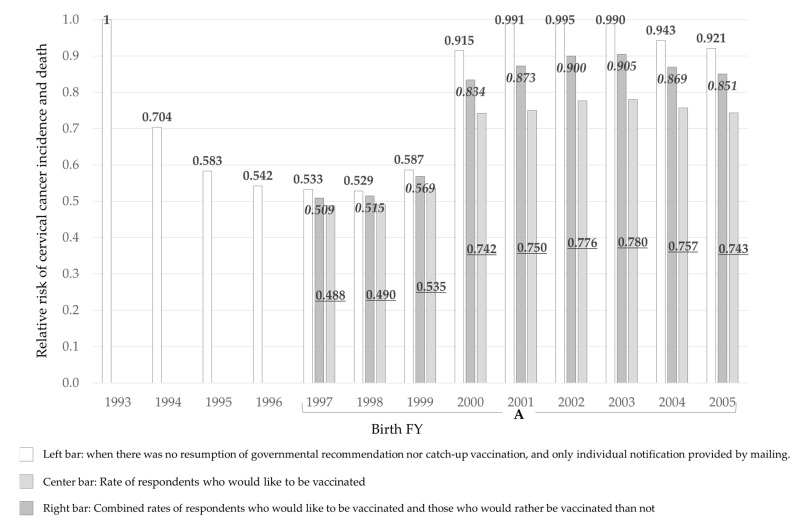
Risks estimated by substituting the vaccination intention obtained from the internet survey as the vaccination rate. A. Targets for catch-up vaccination targets FY2022 to 2024.

In our internet survey of women eligible for catch-up vaccination in FY2022, 13.6% to 25.7% responded that they would like to be vaccinated in FY2022. Using their ‘vaccination intention’ as a substitute for actual future vaccinations, we estimated that the risks for females born in FY1997-99 were 0.509 to 0.569, and those for FY2000-3 were 0.834 and 0.905 (Figure 3). The risks for females born in FY2004-5 were 0.869 and 0.851, respectively. For the two survey groups that responded that they would either ‘definitely like to be vaccinated’ or would ‘rather be vaccinated than not’, the risks for females born in FY1997-99 were 0.488 to 0.535; for FY2000-3, 0.742 to 0.780, and for FY2004-5, 0.757 and 0.743, respectively.

### 3.8. Estimated Number of HPV Vaccine Doses Required, Based on HPV Vaccination Intentions

Under a scenario where catch-up vaccinations are concentrated in FY 2022, 3,131,634 catch-up doses will be needed in FY2022 just for females born in FY1997 to FY2010, based on estimates only for the group that responded that they would like to be vaccinated now (Supplementary Table S1). When the groups that responded that they would ‘definitely like to be vaccinated’ are aggregated with those who would ‘rather be vaccinated than not’, a total of 7,511,061 doses are estimated to be needed in FY2022 for females born from FY1997 to FY2010.

## 4. Discussion

A substantial reduction in cervical cancer in young women after the introduction of the HPV immunization program in Denmark and Sweden [12,13]. A previous study by Brisson M et al. suggested that high HPV vaccination coverage of girls can lead to cervical cancer elimination in most lower-middle-income countries by the end of the century [14]. Screening with high uptake will expedite reductions and will be necessary to eliminate cervical cancer in countries with the highest burden. A systematic review and meta-analysis indicated that an HPV catch-up vaccination could be beneficial; however, the long-term effect of such a vaccination, and its effect on cervical cancer incidence and mortality, is still unclear [15]. Differences in HPV vaccine coverage are observed even among high income countries, reflecting differences in delivery settings. The factors that encourage success often include strong support from government and healthcare organizations, as well as tailored, culturally-appropriate local approaches to optimize outcomes [16].

In the present study, we report that estimates of the relative risk of future cervical cancers and deaths will depend on the cadence and penetration of both routine and catch-up HPV vaccinations. Japan has hopes that its catch-up vaccination program will be somewhat effective in preventing future HPV infections for females already over the vaccine-targeted ages of 12–16. However, to reduce the risk to the same level or lower than that of women born in 1994–1999, catch-up immunization coverage among the unvaccinated must reach as high as 90% by FY2022, when the program begins; even if achieved in FY2024, it will not reduce the risk to the same level as the vaccinated generation.

We had previously calculated the dramatically increased risk of HP-16/18 infection by age 20 due to the catastrophic decrease in HPV vaccination rates caused by the suspension of the MHLW’s recommendation for the vaccine [7]. We reported that the risk of infection logically varied by birth FY, noting that the increase in risk would be minimal if the recommendation had resumed in FY2016. We estimated that each one-year delay in resumption would lead to an increase of about 4000 more cervical cancer cases and 1000 deaths [8]. 

Others have reached the same conclusions. In 2020, Simms et al. proposed a scenario in which vaccination coverage would recover in 2020 and estimated the number of preventable cervical cancer cases and deaths accordingly [17]. Although Yagi and Simms used different estimation methods, the conclusions on excess incidence and death were surprisingly similar. In FY2020, the MHLW issued a nationwide notice that local governments should begin sending ‘HPV vaccine information’ to individually targeted females and parents, and finally, in FY2022, ‘active vaccination recommendation’ was restarted, and ‘catch-up vaccinations’ would be offered for three years. Using updated data of HPV vaccination rates, we estimated the relative lifetime risk for cervical cancer incidence and death by a woman’s FY of birth. We evaluated how much their risk was likely to be reduced by a resumption of the vaccine recommendation and by implementing a catch-up vaccination program using multiple hypothetical values for vaccination coverage to understand the expectations and limitations of catch-up vaccinations.

Our present study updates the report by Yagi A et al. [8], using the latest available vaccination rates, and adding the new reality that the Japanese government has finally decided to resume its recommendation for HPV vaccination and initiate mitigation measures that include catch-up vaccinations. This situation is unique worldwide, so its effects must be studied carefully. Our survey estimated the catch-up vaccination rate would be 13.6% to 25.7% in reality. It seems very difficult to reduce the risk of the females already over the vaccine-targeted ages of 12–16 to the same level or lower than that of women born in 1994–1999. 

The void left by the suspension of the governmental recommendation of HPV vaccine cannot be filled by catch-up vaccination alone. Further measures need to be taken to mitigate the impact of the policy of suspending HPV vaccine recommendations. Along with achieving high immunization coverage for routine and catch-up vaccinations, it will also be important to introduce the nine-valent as the new routine vaccine. Public health impact and cost-effectiveness of catch-up 9-valent HPV vaccination was demonstrated in a previous study [18].

We also need to begin routinely immunizing boys, and more frequently provide accurate information for the media for distribution, and do so in partnership with multiple health agencies. For women whose vaccination coverage waned because of their birth year, strong recommendations for screening should be implemented to achieve cervical cancer prevention and provide the earliest possible treatments. 

## 5. Conclusions

In Japan, where the official recommendation of the vaccine was suspended for almost nine years, it is likely to be impossible to retroactively prevent all the cervical cancer cases and deaths resulting from the vaccination void. We have reported that women from the vaccine-suspension generation are having increasing infection rates with the high-risk HPV types 16 and 18, and are seeing increased rates of abnormal cervical cytology at ages 20 and 21 during their routine cervical screenings [19,20]. It was proposed that a vigorous catch-up vaccination program could alleviate the problem of increased risk to some extent, but there have been limitations already encountered. The public’s and health officials’ confidence in the efficacy and safety of HPV vaccines in Japan is sharply lower than in other countries, with vaccine hesitancy being a major issue. The MHLW and local governments need to take leadership roles to re-popularize the HPV vaccine to compensate.

## Figures and Tables

**Table 1:** The events in Japan regarding the HPV vaccine and its target population.

			1			2							3	4	5		
		FY	2010	2011	2012	2013	2014	2015	2016	2017	2018	2019	2020	2021	2022	2023	2024
	Birth FY																
A	1993		17	18	19	20	21	22	23	24	25	26	27	28	29	30	31
B	1994		16	17	18	19	20	21	22	23	24	25	26	27	28	29	30
	1995		15	16	17	18	19	20	21	22	23	24	25	26	27	28	29
	1996		14	15	16	17	18	19	20	21	22	23	24	25	26	27	28
	1997		13	14	15	16	17	18	19	20	21	22	23	24	25	26	27
	1998		12	13	14	15	16	17	18	19	20	21	22	23	24	25	26
	1999		11	12	13	14	15	16	17	18	19	20	21	22	23	24	25
C	2000		10	11	12	13	14	15	16	17	18	19	20	21	22	23	24
	2001		9	10	11	12	13	14	15	16	17	18	19	20	21	22	23
	2002		8	9	10	11	12	13	14	15	16	17	18	19	20	21	22
	2003		7	8	9	10	11	12	13	14	15	16	17	18	19	20	21
	2004		6	7	8	9	10	11	12	13	14	15	16	17	18	19	20
	2005		5	6	7	8	9	10	11	12	13	14	15	16	17	18	19
D	2006		4	5	6	7	8	9	10	11	12	13	14	15	16	17	18
	2007		3	4	5	6	7	8	9	10	11	12	13	14	15	16	17
	2008		2	3	4	5	6	7	8	9	10	11	12	13	14	15	16
	2009		1	2	3	4	5	6	7	8	9	10	11	12	13	14	15
	2010		0	1	2	3	4	5	6	7	8	9	10	11	12	13	14
	2011			0	1	2	3	4	5	6	7	8	9	10	11	12	13
	2012				0	1	2	3	4	5	6						12

Bold line frame: Eligibility for subsidies and national immunization programs; Cells in gray: Eligibility for catch-up vaccination. Major policy makings related to HPV vaccine: 1. November 2010: Subsidies from local and national governments commenced for an HPV vaccination program for girls who were 7th to 10th-grade students; 2. April 2013: The national immunization program for girls who were 6th to 10th-grade students. June 2013: The MHLW announced the suspension of the governmental recommendation (Notification by the Director-General of the Health Service Bureau of the MHLW). 3. October 2020: The MHLW requested local governments to thoroughly inform those who are eligible for routine vaccinations so that they could consider HPV vaccination. (Notification by the Director-General of the Health Service Bureau of the MHLW). 4. January 2021: The MHLW again requested that local governments thoroughly inform those who were eligible for routine vaccinations (Notification by the Director-General of the Health Service Bureau of the MHLW). November 2021: The MHLW announced the abolition of the suspension of the governmental recommendation. 5. April 2022: The substantive resumption of the governmental recommendation and the start of catch-up vaccination. Status by birth FY: A. Pre-introduction generation, B. Vaccination generation, C. Vaccine-suspension generation, D. Re-introduction generation; Bold line frame: Eligibility for subsidies and national immunization programs; Cells in gray: Eligibility for catch-up vaccination. FY: fiscal year.

**Table 2:** Estimated future incidence and deaths depending on the HPV vaccination rate reached in and after FY2022.

		Birth FY														
	Number *	1997	1998	1999	2000	2001	2002	2003	2004	2005	2006	2007	2008	2009	2010	Total
Where no strategies are conducted against suspension of the governmental recommendation, compared to females born in FY1999 (Assumed the risks of females who born in FY2003 continued for females who born after FY2004)																
	Patients	−592	−634	0	3600	4437	4484	4428	4428	4428	4428	4428	4428	4428	4428	46,719
	Deaths	−157	−169	0	958	1181	1193	1178	1178	1178	1178	1178	1178	1178	1178	12,431
Where only individual notification is conducted form FY2020, compared to females born in FY1999 (Assumed the risks of females who born in FY2008 continued for females who born after FY2009)																
	Patients	−592	−634	0	3600	4437	4484	4428	3910	3660	3487	3385	3311	3311	3311	40,099
	Deaths	−157	−169	0	958	1181	1193	1178	1040	974	928	901	881	881	881	10,669
The assumed vaccination rate **																
Where the routine and catch-up vaccinatios spread evenly through 3 years from FY2022 to 2024, in addition to individual notification																
10%	Patients	−40	−42	−64	3424	4200	4246	4124	3578	3286	3545	3650	3693	3810	3882	41,290
	Deaths	−11	−11	−17	911	1118	1130	1097	952	874	943	971	983	1014	1033	10,986
30%	Patients	−120	−126	−193	3069	3727	3769	3520	2914	2515	2542	2504	3693	3810	3882	35,508
	Deaths	−32	−33	−51	817	992	1003	937	775	669	676	666	983	1014	1033	9448
50%	Patients	−199	−209	−321	2715	3254	3178	2916	2250	1745	1540	1358	2461	2525	2570	25,783
	Deaths	−53	−56	−85	722	866	846	776	599	464	410	361	655	672	684	6860
70%	Patients	−279	−293	−449	2361	2781	2655	2312	1585	974	538	213	1230	1240	1258	16,126
	Deaths	−74	−78	−120	628	740	707	615	422	259	143	57	327	330	335	4291
90%	Patients	−359	−377	−578	2006	2308	2133	1709	921	204	−465	−933	−1233	−1330	−1367	2641
	Deaths	−96	−100	−154	534	614	568	455	245	54	−124	−248	−328	−354	−364	703
Where routine and catch-up vaccination are concentrated in FY2022, in addition to individual notification																
10%	Patients	−115	−116	−169	3135	3903	3946	3893	3422	3193	3473	3610	3680	3802	3880	39,539
	Deaths	−31	−31	−45	834	1038	1050	1036	911	850	924	961	979	1012	1032	10,520
50%	Patients	−574	−578	−843	1274	1767	1795	1762	1471	1327	1182	1162	1167	1202	1245	13,360
	Deaths	−153	−154	−224	339	470	478	469	392	353	315	309	311	320	331	3555
90%	Patients	−1033	−1040	−1517	−585	−369	−356	−369	−479	−540	−1109	−1287	−1345	−1398	−1390	−12,819
	Deaths	−275	−277	−404	−156	−98	−95	−98	−128	−144	−295	−342	−358	−372	−370	−3411

Bold lines: where the number of patients and deaths turn to decline. * The excess cases of incidence and death in each birth FY. ** The assumed vaccination rate means the vaccination rate for non-vaccinated females in FY2022 in each case.

## Data Availability

The datasets generated and/or analyzed during the current study are available from the corresponding author on reasonable request.

## Supplementary Materials

The following supporting information can be downloaded at: https://www.mdpi.com/article/10.3390/vaccines10091455/s1, Table S1: Relative lifetime risk of cervical cancer calculation method; Table S2: Details of cumulative initial HPV vaccination rate by birth FY; Table S3: Estimated number of HPV vaccine doses required based on HPV vaccination intentions.

## Abbreviations

FY: fiscal year, HPV: human papillomavirus, WHO: World Health Organization, MHLW: Ministry of Health, Labor and Welfare.
