# Supplementary material for: Can Catch-Up Vaccinations Fill the Void Left by Suspension of the Governmental Recommendation of HPV Vaccine in Japan?
Source: Vaccines (Basel). 2022 Sep 2;10(9):1455. doi: 10.3390/vaccines10091455 (PMC9502134; doi:10.3390/vaccines10091455)
Supplement: Supplementary file 1 [file vaccines-10-01455-s001.zip › vaccines-1859858-supplementary.pdf]

Supplementary Table S1. Relative lifetime risk of cervical cancer calculation method.

When the vaccination rates of catch-up and routine vaccination spread at once and reaches 50% in FY2022.

Birth FY	Target number								
1997	589055	Age	12	13	14	15	16	25	(Lifetime)
		Number of vaccinations		60149	345946	52608	5722		
		Cumulative vaccination rate		10.20%	68.94%	77.87%	78.84%	89.42%	89.42%
		Sexual experience rate	0%	1%	2%	5%	15%	83%	85%

$$0.4+0.6\times\frac{(1-0.1021)\times(0.01-0.00)+(1-0.6894)\times(0.02-0.01)+(1-0.7787)\times(0.05-0.02)+(1-0.7884)\times(0.15-0.05)+(1-0.8942)\times(0.83-0.15)+(1-0.8942)\times(0.85-0.83)}{1\times0.85}$$

=0.480

Supplementary Table S2. Details of cumulative initial HPV vaccination rate by birth FY.

Birth FY	Target number		2010	2011	2012	2013	2014	2015	2016	2017	2018	2019	2020	2021	2022	2023	2024	2025	2026	Cumulative number of first doses	Cumulative primary vaccination rate
1994	579098	Age	16	17																	
		Number of vaccinations	178852	142613																321465	55.5%
		Vaccination rate	30.9%	24.6%																	
1995	588260	Age	15	16	17																
		Number of vaccinations	64387	362112	5855															432354	73.5%
		Vaccination rate	11.0%	61.6%	1.00%																
1996	589312	Age	14	15	16																
		Number of vaccinations	70362	351268	39224															460854	78.2%
		Vaccination rate	11.9%	59.6%	6.66%																
1997	589055	Age	13	14	15	16															
		Number of vaccinations	60149	345946	52608	5722														464425	78.8%
		Vaccination rate	10.2%	58.7%	8.93%	0.97%															
1998	584594	Age	12	13	14	15	16														
		Number of vaccinations	4364	380434	71102	3838	272													460010	78.7%
		Vaccination rate	0.75%	65.1%	12.2%	0.66%	0.05%														
1999	573656	Age		12	13	14	15	16													
		Number of vaccinations		6836	374526	13198	614	256												395430	68.9%
		Vaccination rate		1.19%	65.3%	2.30%	0.11%	0.04%													
2000	565541	Age			12	13	14	15	16												
		Number of vaccinations			6746	70888	1344	1112	560											80650	14.3%
		Vaccination rate			1.19%	12.5%	0.24%	0.20%	0.10%												
2001		Age				12	13	14	15	16											
	553103	Number of vaccinations				5010	1413	294	534	1366										8617	1.6%
		Vaccination rate				0.91%	0.26%	0.05%	0.10%	0.25%											
2002	543086	Age					12	13	14	15	16										
		Number of vaccinations					236	857	154	800	2614									4661	0.9%
		Vaccination rate					0.04%	0.16%	0.03%	0.15%	0.48%										
2003	529073	Age						12	13	14	15	16									
		Number of vaccinations						178	490	384	1668	6774								9494	1.8%
		Vaccination rate						0.03%	0.09%	0.07%	0.32%	1.28%									
2004	511899	Age							12	13	14	15	16								
		Number of vaccinations							96	663	792	3944	46135							51630	10.1%
		Vaccination rate							0.02%	0.13%	0.15%	0.77%	9.01%								
2005	522030	Age								12	13	14	15	16							
		Number of vaccinations								134	1334	1838	22575	47047						72928	14.0%
		Vaccination rate								0.03%	0.26%	0.56%	4.32%	9.01%							

2006	521772	Age	12	13	14	15							
		Number of vaccinations	402	3833	12883	22563	47024					86705	16.6%
		Vaccination rate	0.08%	0.73%	2.47%	4.32%	9.01%						
2007	521349	Age		12	13	14	15	16					
		Number of vaccinations		908	11377	12872	22545	46986				94688	18.2%
		Vaccination rate		0.17%	2.18%	2.47%	4.32%	9.01%					
2008	510114	Age			12	13	14	15	16				
		Number of vaccinations			6644	11132	12594	22059	45973			98402	19.3%
		Vaccination rate			1.30%	2.18%	2.47%	4.32%	9.01%				
2009	507700	Age				12	13	14	15	16			
		Number of vaccinations				6613	11079	12535	21955	45756		97938	19.3%
		Vaccination rate				1.30%	2.18%	2.47%	4.32%	9.01%			
2010	502411	Age					12	13	14	15	16		
		Number of vaccinations					6544	10963	12404	21726	45279	96916	19.3%
		Vaccination rate					1.30%	2.18%	2.47%	4.32%	9.01%		

Supplementary Table S3. Estimated number of HPV vaccine doses required based on HPV vaccination intentions.

	The respondents who would like to be vaccinated			The respondents who would like to be vaccinated and those who would rather be vaccinated than not		
Birth FY	2022	2023	2024	2022	2023	2024
1997	85,305			161,535		
1998	50,801			136,075		
1999	62,293			179,091		
2000	275,399			593,168		
2001	396,470			808,800		
2002	313,646			721,385		
2003	272,401			673,435		
2004	227,900			576,453		
2005	222,371			562,467		
2006	238,705			603,783		
2007	245,687			621,444		
2008	243,779			616,618		
2009	248,111			627,575		
2010	248,767			629,233		
2011		243,780			616,623	
2012			239,091			604,761
The sum for each FY	3,131,634	243,780	239,091	7,511,061	616,623	604,761

FY: fiscal year
